# Supplementary material for: Inferring feature importance with uncertainties with application to large genotype data
Source: PLoS Comput Biol. 2023 Mar 14;19(3):e1010963. doi: 10.1371/journal.pcbi.1010963 (PMC10038287; doi:10.1371/journal.pcbi.1010963)
Supplement: S1 File — (PDF) [file pcbi.1010963.s001.pdf]

Supporting information

Inferring feature importance with uncertainties with application to large genotype data

Pål Vegard Johnsen*[1,2], Inga Strümke[3,4], Mette Langaas[2], Andrew Thomas DeWan[5], Signe Riemer-Sørensen[1]

1 SINTEF DIGITAL, Oslo, Norway
2 Department of Mathematical Sciences, Norwegian University of Science and Technology, Trondheim, Norway
3 Department of Engineering Cybernetics, Norwegian University of Science and Technology, Trondheim, Norway
4 Department of Holistic Systems, SimulaMet, Oslo, Norway
5 Department of Chronic Disease Epidemiology and Center for Perinatal, Pediatric and Environmental Epidemiology, Yale School of Public Health, New Haven, Connecticut, USA

*pal.johnsen@sintef.no

The weights in Sub-SAGE. The Sub-SAGE, ψ_k, is defined as in Equation 10 in the paper and repeated here for convenience,

$$\psi_k(\mathbf{X}, Y, \hat{y}) = \sum_{\mathcal{S}\in\mathcal{Q}_k} \frac{|\mathcal{S}|!(M-|\mathcal{S}|-1)!}{3(M-1)!} \left[w_{\mathbf{X},Y,\hat{y}}(\mathcal{S}\cup\{k\}) - w_{\mathbf{X},Y,\hat{y}}(\mathcal{S})\right], \tag{1}$$

with $\mathcal{Q}_k$ consisting of the subsets $\{\emptyset\}$, $\{m\}$ for $m = 1, \ldots, k-1, k+1, \ldots, M$ and $\{1, 2, \ldots, k-1, k+1, \ldots, M\}$. In other words, there are three different achievable subset sizes, namely of size zero, one and $M-1$. As we want the sum of all weights to be equal to one, and that the sum of the weights of equal subset size is the same for all subset sizes, we need the corresponding weight for $\mathcal{S} = \{\emptyset\}$ and $\mathcal{S} = \{1, 2, \ldots, k-1, k+1, \ldots, M\}$ to be 1/3, while the sum of the weights for $\mathcal{S} = \{m\}$ for $m = 1, \ldots, k-1, k+1, \ldots, M$ needs to be 1/3. For $\mathcal{S} = \{\emptyset\}$, we see that the weight is $0!(M-1)!/3(M-1)! = 1/3$ and for $\mathcal{S} = \{1, 2, \ldots, k-1, k+1, \ldots, M\}$ the weight is $(M-1)!0!/3(M-1)! = 1/3$, just as we wanted. For the subsets of size one, the weight is $1!(M-2)!/3(M-1)! = 1/3(M-1)$. There are $M-1$ subsets of size one in total, and so the sum of the weights is also 1/3. In other words, the definition of the weights in Sub-SAGE makes sure that the sum of all weights is equal to one, and that the sum of the weights of equal subset size is the same for all subset sizes.

Derivation of Sub-SAGE for squared error and binary cross-entropy. Using as loss function the squared error loss, the loss per sample is $\ell = (y - \hat{y})^2$. Considering a feature k for which to compute the Sub-SAGE value, we separate the trees in our ensemble model into two groups: τ_k, being the set of trees including feature k as a

splitting point, and its complement group ($\bar{\tau}_k$). Then, for any $\mathcal{S} \in \mathcal{Q}_k$,

$$\begin{aligned}
&w_{\mathbf{X},Y,\hat{y}}(\mathcal{S} \cup \{k\}) - w_{\mathbf{X},Y,\hat{y}}(\mathcal{S}) \\
&= E_{\mathbf{X},Y}\left[\left(Y(\mathbf{X}) - V_{\mathbf{X},\hat{y}}(\mathcal{S})\right)^2\right] - E_{\mathbf{X},Y}[\left(Y(\mathbf{X}) - V_{\mathbf{X},\hat{y}}(\mathcal{S} \cup \{k\})\right)^2] \\
&= E_{\mathbf{X},Y}\left[\left(Y - \sum_{j\in\tau_k} V_{\mathbf{X},f_j}(\mathcal{S}) - \sum_{j\notin\tau_k} V_{\mathbf{X},f_j}(\mathcal{S})\right)^2 - \left(Y - \sum_{j\in\tau_k} V_{\mathbf{X},f_j}(\mathcal{S}\cup\{k\}) - \sum_{j\notin\tau_k} V_{\mathbf{X},f_j}(\mathcal{S}\cup\{k\})\right)^2\right] \\
&= E_{\mathbf{X},Y}\left[\left(Y - \sum_{j\in\tau_k} V_{\mathbf{X},f_j}(\mathcal{S}) - \sum_{j\notin\tau_k} V_{\mathbf{X},f_j}(\mathcal{S})\right)^2 - \left(Y - \sum_{j\in\tau_k} V_{\mathbf{X},f_j}(\mathcal{S}\cup\{k\}) - \sum_{j\notin\tau_k} V_{\mathbf{X},f_j}(\mathcal{S})\right)^2\right] \\
&= E_{\mathbf{X},Y}\left[2Y\left(\sum_{j\in\tau_k} V_{\mathbf{X},f_j}(\mathcal{S}\cup\{k\}) - V_{\mathbf{X},f_j}(\mathcal{S})\right) + \left(\sum_{j\in\tau_k} V_{\mathbf{X},f_j}(\mathcal{S})\right)^2 - \left(\sum_{j\in\tau_k} V_{\mathbf{X},f_j}(\mathcal{S}\cup\{k\})\right)^2\right. \\
&\left. + 2\left(\sum_{j\notin\tau_k} V_{\mathbf{X},f_j}(\mathcal{S})\right)\left(\sum_{j\in\tau_k} V_{\mathbf{X},f_j}(\mathcal{S}\cup\{k\}) - V_{\mathbf{X},f_j}(\mathcal{S})\right)\right],
\end{aligned} \tag{2}$$

having used that the two random variables $V_{\mathbf{X},f_j}(\mathcal{S} \cup \{k\})$ and $V_{\mathbf{X},f_j}(\mathcal{S})$ are equivalent, or equal in distribution, for $j \notin \tau_k$. Note that the corresponding observed value $v_{\mathbf{x},f_j}(\mathcal{S} \cup \{k\}) = E_{\mathbf{X}_{\overline{\mathcal{S}}}}[f_j(\mathbf{X}|\mathbf{X}_{\mathcal{S}} = \mathbf{x}_{\mathcal{S}\cup\{k\}})] = E_{\mathbf{X}_{\overline{\mathcal{S}}}}[f_j(\mathbf{X}|\mathbf{X}_{\mathcal{S}} = \mathbf{x}_{\mathcal{S}})] = v_{\mathbf{x},f_j}(\mathcal{S})$ for all $\mathcal{S} \in \mathcal{Q}_k$ since the regression tree f_j does not include feature k, and the features are assumed mutually independent.

Using as loss function the binary cross-entropy, the loss function per sample is

$$\ell = -y \log \hat{y} - (1-y)\log(1-\hat{y}) = (1-y)\sum_{\tau=1}^{T} f_\tau + \log\left(1 + e^{-\sum_{\tau=1}^{T} f_\tau}\right).$$

We then have

$$w_{\mathbf{X},Y,\hat{y}}(\mathcal{S} \cup \{k\}) - w_{\mathbf{X},Y,\hat{y}}(\mathcal{S}) \tag{3}$$

$$= E_{\mathbf{X},Y}\left[(1 - Y(\mathbf{X}))\sum_{\tau=1}^{T} V_{\mathbf{X},f_\tau}(\mathcal{S}) + \log\left(1 + \exp\left(-\sum_{\tau=1}^{T} V_{\mathbf{X},f_\tau}(\mathcal{S})\right)\right)\right] \tag{4}$$

$$- E_{\mathbf{X},Y}\left[(1 - Y(\mathbf{X}))\sum_{\tau=1}^{T} V_{\mathbf{X},f_\tau}(\mathcal{S}\cup\{k\}) + \log\left(1 + \exp\left(-\sum_{\tau=1}^{T} V_{\mathbf{X},f_\tau}(\mathcal{S}\cup\{k\})\right)\right)\right] \tag{5}$$

$$= E_{\mathbf{X},Y}\left[(1 - Y(\mathbf{X}))\left(\sum_{j\in\tau_k} V_{\mathbf{X},f_j}(\mathcal{S}) + \sum_{j\notin\tau_k} V_{\mathbf{X},f_j}(\mathcal{S})\right)\right] \tag{6}$$

$$+ E_{\mathbf{X},Y}\left[\log\left(1 + \exp\left(-\sum_{j\in\tau_k} V_{\mathbf{X},f_j}(\mathcal{S}) - \sum_{j\notin\tau_k} V_{\mathbf{X},f_j}(\mathcal{S})\right)\right)\right] \tag{7}$$

$$- E_{\mathbf{X},Y}\left[(1 - Y(\mathbf{X}))\left(\sum_{j\in\tau_k} V_{\mathbf{X},f_j}(\mathcal{S}\cup\{k\}) + \sum_{j\notin\tau_k} V_{\mathbf{X},f_j}(\mathcal{S}\cup\{k\})\right)\right] \tag{8}$$

$$- E_{\mathbf{X},Y}\left[\log\left(1 + \exp\left(-\sum_{j\in\tau_k} V_{\mathbf{X},f_j}(\mathcal{S}\cup\{k\}) - \sum_{j\notin\tau_k} V_{\mathbf{X},f_j}(\mathcal{S}\cup\{k\})\right)\right)\right] \tag{9}$$

$$= E_{\mathbf{X},Y}\left[(1 - Y(\mathbf{X}))\left(\sum_{j\in\tau_k} V_{\mathbf{X},f_j}(\mathcal{S}) - V_{\mathbf{X},f_j}(\mathcal{S}\cup\{k\})\right)\right. \tag{10}$$

$$+\log\left(\frac{1+\exp\left(-\sum_{j\in\tau_k}V_{\mathbf{X},f_j}(\mathcal{S})-\sum_{j\notin\tau_k}V_{\mathbf{X},f_j}(\mathcal{S})\right)}{1+\exp\left(-\sum_{j\in\tau_k}V_{\mathbf{X},f_j}(\mathcal{S}\cup\{k\})-\sum_{j\notin\tau}V_{\mathbf{X},f_j}(\mathcal{S}\cup\{k\})\right)}\right)\Bigg]. \quad (11)$$

(Sub-)SAGE with multiple linear regression. Consider a fitted linear regression model $\hat{y}_i=\hat{\boldsymbol{\beta}}^T\mathbf{x}_i$, with uncorrelated features. By applying the squared error loss, and by considering $\hat{\boldsymbol{\beta}}$ as a constant (by using data not used to estimate $\hat{\boldsymbol{\beta}}$), we have for a feature k, and a subset $\mathcal{S}\in\mathcal{Q}_k$ that

$$\begin{aligned}
&w_{\mathbf{X},Y,\hat{y}}(\mathcal{S}\cup\{k\})-w_{\mathbf{X},Y,\hat{y}}(\mathcal{S})=E_{\boldsymbol{X},Y}[(Y-V_{\boldsymbol{X},\hat{y}}(\mathcal{S}))^2]-E_{\boldsymbol{X},Y}[(Y-V_{\boldsymbol{X},\hat{y}}(\mathcal{S}\cup\{k\}))^2]\\
&=E_{\mathbf{X},Y}[2Y\hat{\beta}_k(X_k-E[X_k])+V_{\boldsymbol{X},\hat{y}}(\mathcal{S})^2-V_{\boldsymbol{X},\hat{y}}(\mathcal{S}\cup\{k\})^2]\\
&=2\hat{\beta}_kE_{\mathbf{X},Y}\left[Y(X_k-E[X_k])\right]+2E_{\mathbf{X},Y}\left[\hat{\beta}_k(\hat{\beta}_S^TX_S+\hat{\beta}^T_{\overline{\mathcal{S}\cup\{k\}}}E[X_{\overline{\mathcal{S}\cup\{k\}}}])(E[X_k]-X_k)\right]\\
&-\hat{\beta}_k^2E_{\mathbf{X},Y}\left[\left({X_k}^2-E[X_k]^2\right)\right]\\
&=2\hat{\beta}_k\operatorname{Cov}(Y,X_k)-\hat{\beta}_k^2\operatorname{Var}(X_k),
\end{aligned} \quad (12)$$

with

$$V_{\boldsymbol{X},\hat{y}}(\mathcal{S})=\hat{\beta}_kE[X_k]+\hat{\beta}_{\mathcal{S}}^T\mathbf{X}_s+\hat{\beta}^T_{\overline{\mathcal{S}\cup\{k\}}}E[\mathbf{X}_{\overline{\mathcal{S}\cup\{k\}}}],$$

the stochastic version of
$v_{\boldsymbol{x},\hat{y}}(\mathcal{S})=E[\hat{y}(\boldsymbol{X})|X_{\mathcal{S}}=x_{\mathcal{S}}]=\hat{\beta}_kE[X_k]+\hat{\beta}_{\mathcal{S}}x_s+\hat{\beta}_{\overline{\mathcal{S}\cup\{k\}}}E[X_{\overline{\mathcal{S}\cup\{k\}}}]$, and

$$V_{\boldsymbol{X},\hat{y}}(\mathcal{S}\cup\{k\})=\hat{\beta}_kX_k+\hat{\beta}_{\mathcal{S}}X_s+\hat{\beta}_{\overline{\mathcal{S}\cup\{k\}}}E[X_{\overline{\mathcal{S}\cup\{k\}}}],$$

the stochastic version of $v_{\boldsymbol{x},\hat{y}}(\mathcal{S}\cup\{k\})$. See Appendix B in [1] for derivation of $v_{\boldsymbol{x},\hat{y}}(\mathcal{S})$ in linear regression. The second term in the third line of Eq. (12) is equal to zero since the features are independent, and $\hat{\boldsymbol{\beta}}$ is considered a constant. Notice therefore that the Sub-SAGE value, as well as the Shapley additive global importance (SAGE)-value, is independent of the subset $\mathcal{S}$ used, and equal to Eq. (12).

The second term ${\hat{\beta}_k}^2\operatorname{Var}(X_k)$ is in fact equal to the increased variance in the model by including feature k actively in the model since

$$\begin{aligned}
&E[V_{\boldsymbol{X},\hat{y}}(\mathcal{S})^2-V_{\boldsymbol{X},\hat{y}}(\mathcal{S}\cup\{k\})^2]\\
&=E[V_{\boldsymbol{X},\hat{y}}(\mathcal{S})^2]-E[V_{\boldsymbol{X},\hat{y}}(\mathcal{S})]^2-(E[V_{\boldsymbol{X},\hat{y}}(\mathcal{S}\cup\{k\})^2]-E[V_{\boldsymbol{X},\hat{y}}(\mathcal{S}\cup\{k\})]^2)\\
&=\operatorname{Var}(V_{\boldsymbol{X},\hat{y}}(\mathcal{S}))-\operatorname{Var}(V_{\boldsymbol{X},\hat{y}}(\mathcal{S}\cup\{k\})),
\end{aligned} \quad (13)$$

because $E[V_{\boldsymbol{X},\hat{y}}(\mathcal{S})]=E[V_{\boldsymbol{X},\hat{y}}(\mathcal{S}\cup\{k\})]$.

For linear regression models, this shows that the Sub-SAGE value is only positive if the agreement between the model and the independent test data (first term in Eq. (12)) upweights the increased variance in the model (second term in Eq. (12)) by including feature k.

We neither know the variance of X_k nor the correlation between X_k and Y, and so these must also be estimated from the data. The sample mean and sample covariance are unbiased and consistent estimators. Therefore, by using *independent* test data $(\mathbf{x}_1^0,y_1^0),...,(\mathbf{x}_{N_I}^0,y_{N_I}^0)$ of size N_I, the estimator of $\hat{\beta}_k$, denote it $T(\hat{\beta}_k)$, is statistically independent from the test data, and by applying the sample mean and covariance we

get the following unbiased estimate of Eq. (12)

$$\begin{aligned}
&\hat{w}_{\mathbf{X},Y,\hat{y}}(\mathcal{S}\cup\{k\}) - \hat{w}_{\mathbf{X},Y,\hat{y}}(\mathcal{S}) \\
&= \frac{2\hat{\beta}_j}{n_I - 1}\sum_{i=1}^{N_I}\left[y_i^0 x_{i,j}^0 - \left(\frac{1}{N_I}\sum_{i=1}^{N_I} x_{i,j}\right)\left(\frac{1}{N_I}\sum_{i=1}^{N_I} y_{i,j}\right)\right] - \hat{\beta}_j{}^2 \frac{1}{N_I - 1}\sum_{i=1}^{N_I}\left(x_{i,j} - \frac{1}{N_I}\sum_{i=1}^{N_I} x_{i,j}\right)^2 \\
&= 2\hat{\beta}_j \widehat{\mathrm{Cov}}^0(Y, X_k) - \hat{\beta}_k{}^2 \widehat{\mathrm{Var}}^0(X_k)\,.
\end{aligned} \tag{14}$$

If we did not use training data separately for constructing the model, and test data to compute Sub-SAGE values, the second term in the third line of Eq. (12) would no longer become zero since the estimator $T(\hat{\boldsymbol{\beta}})$ naturally is correlated with the training data itself. It may seem confusing to treat $\hat{\beta}_k$ in Eq. (12) as a constant when the corresponding estimator $T(\hat{\beta}_k)$ indeed has a distribution based on the training data. However, one may look at the procedure of Sub-SAGE as objectively observing the properties of the raw model itself without taking into account the data used for training the model.

Sub-SAGE estimate for tree ensemble models with tree stumps. Consider a tree ensemble model with regression trees of depth one, so-called tree stumps. Each tree stump includes exactly one feature from the set $\mathcal{M}$ of all M features. In accordance with earlier notation, let τ_k denote the set of tree stumps that include feature k. Then, Eq. (2) reduces to

$$\begin{aligned}
&w_{\mathbf{X},Y,\hat{y}}(\mathcal{S}\cup\{k\}) - w_{\mathbf{X},Y,\hat{y}}(\mathcal{S}) \\
&= E_{\mathbf{X},Y}\Bigg[2Y\left(\sum_{j\in\tau_k} V_{\mathbf{X},f_j}(\mathcal{S}\cup\{k\}) - V_{\mathbf{X},f_j}(\mathcal{S})\right) + \left(\sum_{j\in\tau_k} V_{\mathbf{X},f_j}(\mathcal{S})\right)^2 - \left(\sum_{j\in\tau_k} V_{\mathbf{X},f_j}(\mathcal{S}\cup\{k\})\right)^2 \\
&+ 2\left(\sum_{j\notin\tau_k} V_{\mathbf{X},f_j}(\mathcal{S})\right)\left(\sum_{j\in\tau_k} V_{\mathbf{X},f_j}(\mathcal{S}\cup\{k\}) - V_{\mathbf{X},f_j}(\mathcal{S})\right)\Bigg] \\
&= 2Cov\left(Y, \sum_{j\in\tau_k} f_j(X_k)\right) - Var\left(\sum_{j\in\tau_k} f_j(X_k)\right)\,,
\end{aligned} \tag{15}$$

because all random variables $V_{\mathbf{X},f_j}(\mathcal{S})$ for $j \notin \tau_k$, for every $\mathcal{S}$ are now independent of all $V_{\mathbf{X},f_j}(\mathcal{S})$ and $V_{\mathbf{X},f_j}(\mathcal{S}\cup\{k\})$ for $j \in \tau_k$. Further, for every $j \in \tau_k$, $V_{\mathbf{X},f_j}(\mathcal{S}) = E_{\mathbf{X}}[f_j(\mathbf{X})]$, a constant equal to the expected value of the output of the regression tree f_j, and $E_{\mathbf{X}}[V_{\mathbf{X},f_j}(\mathcal{S}\cup\{k\})] = E_{\mathbf{X}}[f_j(\mathbf{X})]$, since the regression tree f_j only includes feature k. Therefore, the last term in Eq. (2) vanishes. Observe that, in the case of tree stumps,

$$\begin{aligned}
&E_{\mathbf{X},Y}\left[Y\left(\sum_{j\in\tau_k} V_{\mathbf{X},f_j}(\mathcal{S}\cup\{k\}) - V_{\mathbf{X},f_j}(\mathcal{S})\right)\right] \\
&= E_{\mathbf{X},Y}\left[Y\left(\sum_{j\in\tau_k} f_j(X_k)\right)\right] - E_Y[Y]E_{\mathbf{X}}\left[\sum_{j\in\tau_k} f_j(X_k)\right] = \mathrm{Cov}\left(Y, \sum_{j\in\tau_k} f_j(X_k)\right)\,.
\end{aligned}$$

Likewise,

$$E_{\mathbf{X},Y}\left[\left(\sum_{j\in\tau_k} V_{\mathbf{X},f_j}(\mathcal{S}\cup\{k\})\right)^2 - \left(\sum_{j\in\tau_k} V_{\mathbf{X},f_j}(\mathcal{S})\right)^2\right]$$
$$= E_{\mathbf{X}}\left[\left(\sum_{j\in\tau_k} f_j(X_k)\right)^2\right] - E_{\mathbf{X}}\left[\sum_{j\in\tau_k} f_j(X_k)\right]^2 = \mathrm{Var}\left(\sum_{j\in\tau_k} f_j(X_k)\right).$$

Hence, the expression given in Eq. (15) independent of the subset $\mathcal{S}$. The expression in Eq. (15) is therefore also equal to the Sub-SAGE value, $\hat{\psi}_k$ (or SAGE value). Both the covariance and the variance need to be must be estimated in practice. Given independent test data $(\mathbf{x}_1^0, y_1^0), \ldots, (\mathbf{x}_{N_I}^0, y_{N_I}^0)$, an unbiased estimate is given by

$$\hat{\psi}_k = \frac{1}{N_I^0 - 1}\sum_{i=1}^{N_I^0}\left(y_i^0 - \sum_{i=1}^{N_I} y_i^0\right)\left(\sum_{j\in\tau_k} f_j(x_{i,k}^0) - \sum_{j\in\tau_k} v_{x_{i,k}^0,f_j}(\emptyset)\right)$$
$$- \frac{1}{N_I^0 - 1}\sum_{i=1}^{N_I^0}\left(\sum_{j\in\tau_k} f_j(x_{i,k}^0) - \sum_{j\in\tau_k} v_{x_{i,k}^0,f_j}(\emptyset)\right)^2 . \tag{16}$$

Sub-SAGE properties related to Shapley values. Symmetry, null player, linearity, monotonicity and efficiency are all properties of Shapley values. Below we investigate whether the same properties apply for Sub-SAGE values.

Property 1 (**Symmetry**)**.** Given two features j and k such that $v(\mathcal{S}\cup\{j\}) = v(\mathcal{S}\cup\{k\})$ for all $\mathcal{S}\in\{\mathcal{Q}_j, \mathcal{Q}_k\}$ in which $\{j,k\}\notin\mathcal{S}$. Then their Sub-SAGE values indeed are identical, $\psi_j = \psi_k$, and so the symmetry property follows by definition. This means in practice that two perfectly correlated features have equal Sub-SAGE values.

Property 2 (**Dummy property (null player)**)**.** Given a feature k where $v(\mathcal{S}\cup\{k\}) = v(\mathcal{S})$ for all $\mathcal{S}\in\mathcal{Q}_k$. Then $\psi_k = 0$, and so the dummy property follows by definition.

Property 3 (**Linearity**)**.** Given two value functions $v(\mathcal{S})$ and $w(\mathcal{S})$, the Sub-SAGE value of the sum of the value functions $v(\mathcal{S}) + w(\mathcal{S})$ is equal to the sum of the Sub-SAGE for each value function,

$$\psi_k(v+w) = \psi_k(v) + \psi_k(w). \tag{17}$$

Hence, the linearity property follows by definition.

Property 4 (**Monotonicity**)**.** Consider two models $\hat{f}_1$ and $\hat{f}_2$ used to predict the same relationship $y = f(\mathbf{x})$, for the same features $\mathbf{x}$. If for any feature k we have $v_{\hat{f}_1}(\mathcal{S}\cup\{k\}) - v_{\hat{f}_1}(\mathcal{S}) \geq v_{\hat{f}_2}(\mathcal{S}\cup\{k\}) - v_{\hat{f}_2}(\mathcal{S})$ for all $\mathcal{S}\in\mathcal{Q}_k$, then by definition, $\psi_k^{\hat{f}_1} \geq \psi_k^{\hat{f}_2}$, with $\psi_k^{\hat{f}_1}$ the Sub-SAGE value of feature k when applying model $\hat{f}_1$ and $\psi_k^{\hat{f}_2}$ the corresponding Sub-SAGE value when applying model $\hat{f}_2$. This means that an adjustment of model $\hat{f}_2$ to $\hat{f}_1$ such that feature k's importance increases also increases its Sub-SAGE value. Therefore, the monotonicity property follows by definition.

Observation 1 (**Sub-SAGE does not share the efficiency property**)**.** Consider the definition of the Shapley value, ϕ_k, applied on a specific value function v:

$$\phi_k = \sum_{\mathcal{S}\subseteq\mathcal{M}\setminus\{k\}} \frac{|\mathcal{S}|!(M - |\mathcal{S}| - 1)!}{M!}\left[v(\mathcal{S}\cup\{k\}) - v(\mathcal{S})\right]. \tag{18}$$

The efficiency property for the Shapley value reads

$$\sum_{k=1}^{M} \phi_k = v(\mathcal{M}) - v(\emptyset)\,, \tag{19}$$

for M "players". This can be observed more easily by using instead the following formulation of the Shapley value

$$\phi_k = \frac{1}{M!} \sum_{R} \left[v(s_k(R) \cup \{k\}) - v(s_k(R)) \right]\,, \tag{20}$$

where the sum is over all *orderings* R of the M features, with a total of $M!$ orders. The function $s_k(R)$ maps a given ordering R and a particular feature k to the corresponding subset of features preceding feature k in the specific ordering. For instance, for $\mathcal{M} = \{1, 2, 3\}$, one possible ordering is $R = (2, 3, 1)$ with $s_1(R) = (2, 3)$. We then have

$$\begin{aligned}
\sum_{k=1}^{M} \phi_k &= \sum_{k=1}^{M} \frac{1}{M!} \sum_{R} \left[v(s_k(R) \cup \{k\}) - v(s_k(R)) \right] \\
&= \frac{1}{M!} \sum_{R} \sum_{k=1}^{M} \left[v(s_k(R) \cup \{k\}) - v(s_k(R)) \right] \\
&= \frac{1}{M!} \sum_{R} \left(v(\mathcal{M}) - v(\emptyset) \right) \\
&= \frac{1}{M!} M! \left(v(\mathcal{M}) - v(\emptyset) \right) = v(\mathcal{M}) - v(\emptyset),
\end{aligned} \tag{21}$$

since for a specific ordering R and feature k, in the sum $\sum_{k=1}^{M} \left[v(s_k(R) \cup \{k\}) - v(s_k(R)) \right]$ all terms cancel each other, except $v(\mathcal{M})$ and $v(\emptyset)$.

The Sub-SAGE value, ψ_k, for a feature k is not a sum over all subsets $\mathcal{S} \subseteq \mathcal{M} \setminus \{k\}$, but limited to the sets in $\mathcal{Q}_k$,

$$\psi_k(\mathbf{y}, \hat{\mathbf{y}}) = \sum_{\mathcal{S} \in \mathcal{Q}_k} \frac{|\mathcal{S}|!(M - |\mathcal{S}| - 1)!}{3(M-1)!} \left[v\left(\mathcal{S} \cup \{k\}\right) - v\left(\mathcal{S}\right) \right]\,, \tag{22}$$

and therefore, from the definition in Eq. (20), is *not* the sum over all orderings R. The Sub-SAGE value therefore does not share the efficiency property of the Shapley value.

SHAP computations for Fig. 3. Consider this time the SHAP value of a given data generating process, f, with known relationship:

$$\phi_k^{\mathrm{SHAP}}(\mathbf{x}, f) = \sum_{\mathcal{S} \subseteq \mathcal{M} \setminus \{k\}} \frac{|\mathcal{S}|!(M - |\mathcal{S}| - 1)!}{M!} \left[v_{\mathbf{x},f}(\mathcal{S} \cup \{k\}) - v_{\mathbf{x},f}(\mathcal{S}) \right], \tag{23}$$

By applying the data generating process, f, explained in Section 5, the exact SHAP value of feature 1 can be computed by partitioning in the subsets $\mathcal{S}$ *not including* feature 2, as well as those *including* feature 2. For all $\mathcal{S}$ not including feature 2, and by using the result in Appendix B in [1]:

$$v_{\mathbf{x}_i,f}(\mathcal{S} \cup \{k\}) - v_{\mathbf{x}_i,f}(\mathcal{S}) = a_1(x_{i,1} - E[X_1]) + a_{21} E[e^{X_2}](x_{i,1} - E[X_1]),$$

independent of the subset $\mathcal{S}$ used. Of all $\mathcal{S} \subseteq \mathcal{M} \setminus \{1\}$, half of them will not include feature 2, and the sum of the corresponding Shapley weights are given by:

$$
\sum_{|\mathcal{S}|=0}^{M-2} \frac{|\mathcal{S}|!(M-|\mathcal{S}|-1}{M!}\binom{M-2}{|\mathcal{S}|} = \sum_{|\mathcal{S}|=0}^{M-2} \frac{|\mathcal{S}|!(M-|\mathcal{S}|-1}{M!}\frac{(M-2)!}{|\mathcal{S}|!(M-2-|\mathcal{S}|!)}
$$

$$
= \sum_{|\mathcal{S}|=0}^{M-2} \frac{1}{M} - \frac{1}{M(M-1)} \sum_{|\mathcal{S}|=0}^{M-2} |\mathcal{S}| = \frac{1}{2}.
$$

For all $\mathcal{S}$ including feature 2:

$$
v_{\mathbf{x}_i,f}(\mathcal{S} \cup \{k\}) - v_{\mathbf{x}_i,f}(\mathcal{S}) = a_1(x_{i,1} - E[X_1]) + a_{21}e^{x_{i,2}}(x_{i,1} - E[X_1]).
$$

As the sum of the Shapley weights are equal to one, the sum of the Shapley weights for these $\mathcal{S}$ must also be 1/2. Hence, the SHAP value of feature 1 is given by:

$$
\begin{aligned}
\phi_{i,1}(\mathbf{x}_i) &= \frac{1}{2}(a_1(x_{i,1} - E[X_1]) + a_{21}E[e^{X_2}](x_{i,1} - E[X_1])) \\
&+ \frac{1}{2}(a_1(x_{i,1} - E[X_1]) + a_{21}e^{x_{i,2}}(x_{i,1} - E[X_1])) \\
&= a_1(x_{i,1} - E[X_1]) + a_{21}E[e^{X_2}](x_{i,1} - E[X_1]) \\
&+ \frac{1}{2}a_{21}x_{i,1}(e^{x_{i,2}} - E[e^{X_2}]) - \frac{1}{2}a_{21}E[X_1](e^{x_{i,2}} - E[e^{X_2}]).
\end{aligned} \tag{24}
$$

In the exact same manner one can show that:

$$
\begin{aligned}
\phi_{i,2}(\mathbf{x}_i) &= \frac{1}{2}(a_2(x_{i,2} - E[X_2]) + a_{21}E[X_1](e^{x_{i,2}} - E[e^{X_2}]) \\
&+ \frac{1}{2}(a_2(x_{i,2} - E[X_2]) + a_{21}x_{i,1}(e^{x_{i,2}} - E[e^{X_2}])) \\
&= a_2(x_{i,2} - E[X_2]) + a_{21}E[X_1](e^{x_{i,2}} - E[e^{X_2}]) \\
&+ \frac{1}{2}a_{21}x_{i,1}(e^{x_{i,2}} - E[e^{X_2}]) - \frac{1}{2}a_{21}E[X_1](e^{x_{i,2}} - E[e^{X_2}]).
\end{aligned} \tag{25}
$$

$$
\begin{aligned}
&\phi_{i,6} = \frac{1}{2}(a_6E[X_5](I(x_{i,6} > 7) - E[I(X_6 > 7)]) \\
&\frac{1}{2}(a_6x_{i,5}(I(x_{i,6} > 7) - E[I(X_6 > 7)])) \\
&= a_6E[X_5](I(x_{i,6} > 7) - E[I(X_6 > 7)]) \\
&+ \frac{1}{2}a_6I(x_{i,6} > 7)(x_{i,5} - E[X_5]) - \frac{1}{2}a_6E[I(X_6 > 7)](x_{i,5} - E[X_5]).
\end{aligned} \tag{26}
$$

(Sub-)SAGE computation of "Proof of concept" example. We will summarize the computations for achieving the Sub-SAGE and SAGE value for the features 6, 1, 2 and 12 for the true model given in (19). First, for squared error loss, we get that:

$$
\begin{aligned}
&w_{\mathbf{X},Y,\hat{y}}(\mathcal{S} \cup \{k\}) - w_{\mathbf{X},Y,\hat{y}}(\mathcal{S}) = \mathrm{E}_{\mathbf{X},Y}[(Y - V_{\mathbf{X},\hat{y}}(\mathcal{S}))^2] - \mathrm{E}_{\mathbf{X},Y}[(Y - V_{\mathbf{X},\hat{y}}(\mathcal{S} \cup \{k\}))^2] \\
&= 2\,\mathrm{E}_{\mathbf{X},Y}[Y(V_{\mathbf{X}}(\mathcal{S} \cup \{k\}) - V_{\mathbf{X},\hat{y}}(\mathcal{S}))] - \mathrm{E}_{\mathbf{X},Y}[V_{\mathbf{X},\hat{y}}(\mathcal{S} \cup \{k\})^2 - V_{\mathbf{X},\hat{y}}(\mathcal{S}))^2]
\end{aligned} \tag{27}
$$

The first expectation term in (27) measures the covariance between the response and change in the model output when including feature k actively in the model. The second expectation is the increased variance of the model. For both SAGE and Sub-SAGE, the difference in (27) for the model in (19) will differ in at most two sets of subsets for any k as there are at most pair-wise interaction effects. For a feature k which is in a pairwise interaction with some other feature m, the difference in (27) will be the same

among all $\mathcal{S}$ such that $\{m\} \notin \mathcal{S}$, and also the same among all $\mathcal{S}$ such that $\{m\} \in \mathcal{S}$. For both SAGE, and Sub-SAGE, let k_1 and k_2 be the sum of the weights for all subsets $\mathcal{S}$ where $\{m\} \notin \mathcal{S}$ and for all subsets $\mathcal{S}$ where $\{m\} \in \mathcal{S}$ respectively. For SAGE, using the results from S6 Appendix, $k_1 = k_2 = 1/2$. For Sub-SAGE, one can similarly show that

$$k_1 = \frac{1}{3} + \frac{(M-2)}{3(M-1)},$$

$$k_2 = \frac{1}{3} + \frac{1}{3(M-1)},$$

where M is the total number of features in the model. In other words, the Sub-SAGE and SAGE value only differ due to these constants for a

As a practical example we will consider feature 6 ($k = 6$). The first term in (27) can be directly derived using the computed SHAP value for feature 6, and swap observed values with random variables to get $V_{\mathbf{X},Y}(\mathcal{S}))$ and $V_{\mathbf{X},\hat{y}}(\mathcal{S} \cup \{k\}))$ instead of $v_{\mathbf{x},\hat{y}}(\mathcal{S}))$ and $v_{\mathbf{x},\hat{y}}(\mathcal{S}))$. Due to the assumption of feature independence, the second term in (27) involving increased variance only depends on features 5 and 6. As a result, we get that the Sub-SAGE value is given by:

$$\begin{aligned}\psi_6(\mathbf{X}, Y, \hat{y}) &= 2\beta_6 \operatorname{E}[X_5] k_1 \operatorname{E}_{\mathbf{X},Y}[Y(I(X_6 > 7) - E[(I(X_6 > 7)])] \\ &+ 2\beta_6 k_2 \operatorname{E}_{\mathbf{X},Y}[Y X_5 (I(X_6 > 7) - E[(I(X_6 > 7)])] \\ &- (k_1 E[X_5]^2 + k_2 E[X_5^2]) \operatorname{Var}(I(X_6 > 7)).\end{aligned} \tag{28}$$

The expected values can be computed exactly by construction. For the corresponding SAGE value, change k_1 and k_2 accordingly. See Table 2 in paper.

References

1. Aas K, Jullum M, Løland A. Explaining individual predictions when features are dependent: More accurate approximations to Shapley values. Artificial Intelligence. 2021;298.
