# Supplementary material for: Inferring feature importance with uncertainties with application to large genotype data
Source: PLoS Comput Biol. 2023 Mar 14;19(3):e1010963. doi: 10.1371/journal.pcbi.1010963 (PMC10038287; doi:10.1371/journal.pcbi.1010963)
Supplement: Attachment — Submitted filename: Review_PlosComp.pdf [file pcbi.1010963.s002.pdf]

Comment on revision for the manuscript "Inferring feature importance with uncertainties with application to high-dimensional genotype data"

We would like to thank the reviewers for their thorough reading of the paper and for providing critical and constructive comments that have led to an overall improvement of our manuscript. We first provide some overall comments replying to questions that were raised by all reviewers before we address the comments from each reviewer. Changes made to the manuscript are given in cyan colour.

Answer to Reviewer 1 (first specific comment), Reviewer 2 and Reviewer 3 (major comments 1 and 2) regarding application of method on high-dimensional data:

All reviewers are, rightfully, questioning about the application of high-dimensional data in the UK Biobank application (< 3,000 single nucleotide polymophisms (SNPs) and 64,000 samples). To answer this, we want to emphasize the close connection between the results in [1] and this paper. In [1], the full data consists of 529 024 SNPs (after quality control) and 207 015 individuals. Here, a method is proposed for detecting the most important SNPs, taking into account non-linear effects, relevant for predicting a particular phenotype using the black-box machine learning model XGBoost combined with SHAP values to quantify the importance of each such SNP. This method can be seen as a filtering procedure for finding a subset of **promising** candidate SNPs. In this paper, we use a particular model constructed from [1], on the **aforementioned** reduced subset of promising SNPs.

However, as the use of SHAP values is a *model-agnostic* method, the feature importance quantity relies on the *model* itself, and is therefore highly sensitive to the performance of the model. We therefore argue for the use of SAGE values instead as it relies on the *loss function, w*hich quantifies how well the model describes the data generating process. Unfortunately, as described in [2], computing SAGE values quickly becomes infeasible even for a moderately sized reduced subset of features in the context of biological data. We therefore introduce sub-SAGE values.

In this sense, we argue that the work presented in this paper is *related* to high-dimensional data since it starts with the analysis of high-dimensional data. We then propose a search method for finding the most important features by first reducing the number of features to consider, for instance as described by [1] when applied to GWAS. As the filtered promising features, possibly of moderate size, need to be inspected more thoroughly, we further propose to use sub-SAGE values as the computation of SAGE values in this case may be computationally infeasible. The number of iterations/subsets required for computing a SAGE value increases *exponentially* with the number of features (for instance a total of 500 features gives 2^500 =3.273391e+150 subsets to compute). Keep in mind that the method presented in this paper is not restricted to genome-wide association studies, but only used GWAS as a typical use-case of high-dimensional biological data. This method, and the arguments and discussions behind, is relevant whenever a black-box machine learning model is applied on high-dimensional biological data, even though we in this case have provided algorithms tailored for XGBoost models.

However, as our intention is not to use sub-SAGE directly in a full-scale GWAS, and high-dimensional data is often defined as the case when $p \approx n$ or $p > n$, we have decided to edit the title of the manuscript by swapping "high-dimensional genotype data" with "large genotype data".

We do agree with the reviewers that our arguments provided above have not been sufficiently clearly explained for the reader. We have therefore made these arguments clearer in the subsection

"Sub-Sage" as well as the subsection "Application on genetic data using the UK Biobank resource". Here, we describe the method in [1] in more detail to clarify that the model we are using from this paper is indeed based on high-dimensional data (around 530 000 SNPs and 210 000 individuals). This is the new text added in the manuscript in the section "Application on genetic data using the UK Biobank Resource" and shown in cyan in the revised manuscript:

"In detail, the particular XGBoost model mentioned above was constructed after the so-called ranking process explained in [1]*, which ranks features by importance and filter for correlation. Using a sample of 207 015 individuals, a total of 529 024 SNPs were split into 50 randomly selected subsets, each consisting of SNPs with mutually small correlation (Pearson's correlation r^2< 0.2) and unrelated individuals, with the purpose of limiting the correlation between features due to linkage disequilibrium* [3]*, as well as reducing the effect of population stratification and cryptic relatedness* [4]*. For each such subset, XGBoost models in combination with cross-validation were fitted, and the importance of each SNP, taking into account all the generated models, was measured according to the model-agnostic ERFC score introduced in* [1] *based on SHAP values. The ranked list of features by importance was again filtered for correlation (Pearson's correlation r^2< 0.2), and used during the so-called model fitting process based on data not used during the ranking process (see Figure 3 in* [1]*). The aim in the model fitting process is to find how large the portion of top ranked features in the training data must be to get the strongest prediction model, using the PR-AUC metric* [5]*, restricted to an ensemble model consisting of XGBoost models constructed via cross-validation (see Figure 9 in* [1]*). From the best performing ensemble model, and for simplicity restricted to regression trees of maximum depth equal to two, we picked one of the XGBoost models from this ensemble model as the particular model to investigate further in this paper."*

We removed the sentence:

"We study a particular XGBoost model constructed in [1] based on 3000 features both genetic SNP and non-genetic, for 64 000 unrelated White-British participants from UK Biobank."

This sentence can be misinterpreted to mean that we only look at 3000 features and 64 000 individuals. However, the 3000 features are based on the so-called *ranking process* introduced in [1] where indeed LD and population stratification are accounted for, and we look at 207 015 individuals and 529 024 SNPs. From the ranking process, the importance of each of these 529 024 SNPs is estimated. Further in the *model fitting process* the aim is to find which portion of the top ranked features that gives the strongest predictive model, where the model in this case is an *ensemble* model consisting of XGBoost models generated during cross-validation. When we restrict to maximum depth of two in the regression trees of the XGBoost models (then interactions beween to inputs can be modelled), the best ensemble model is when the top 3000 features are included (see Figure 9a in [1]), hence the 3000 features we were referring to in the previous manuscript. Regarding the 64 000 individuals, these refer to the number of individuals that is used to train a particular XGBoost model in a 4-fold cross-validation. The *fitting data* that is used during the model fitting process included 80 000 individuals, where one fifth (16 000) where used as left-out test data to evaluate the ensemble models. We decided to not include the specific information given in this paragraph in the manuscript, but rather inform the reader as concise as possible how the XGBoost model was constructed and how linkage disequilibrium (leading to correlations) and population stratification (which may lead to spurious effects due to confounders) were accounted for.

Answer to Reviewer 1 (second question regarding UK Biobank example), and Reviewer 3 (major question 1) regarding correlations in the data:

In [1], correlation between SNPs via *linkage disequilibrium* (LD) was accounted for during the proposed *ranking process* and *model fitting process* **leading to the** XGBoost model that we have used in the current manuscript. Consequently, sub-SAGE computations are based on features with low mutual correlation. If there are significant correlations in the data **to be used** when fitting a model, conditional expectations need to be estimated, as we discuss towards the end of the paper, concluding that this is often too computationally heavy.

We have now in detail explained how LD was accounted for in the subsection "Application to genetic data using the UK Biobank resource" as we agree with the reviewers this was not sufficiently well explained.

Reviewer 1:

Comment: The high-dimensional example provided is still a relatively small dataset given the current size of GWAS studies - can the authors provide an indication of run-time to estimate the sub-SAGE values?

Answer: See comment above to all reviewers.

Comment: the authors propose selecting the reduced number of features based on either domain knowledge or by "selecting the top tier". In the case of the former, might this limit the ability for discovery of novel genetic relationships? For the latter, there is no indication as to what this entails - some further details would be helpful.

Answer: Regarding the former, we agree that this limits the objective ability for discovery, and have removed this specific sentence. Regarding the latter, we have elaborated as to how this can be done, for instance by using the procedure proposed in [1], and the limitations of the approach. See edits highlighted in cyan colour in the subsection "Application on genetic data using the UK Biobank resource".

Comment: Have the XGBoost hyperparameters mentioned been optimised?

Answer: Yes, on a reduced hyperparameter set. See edited description in the subsection "Application on genetic data using the UK Biobank resource".

Comment: Is there any attempt to account for correlation in SNP importance, based on e.g. LD structure and if not, how does this affect importance estimates?

Answer: Yes, see comment above regarding correlation in data.

https://www.nature.com/articles/s42003-022-03812-z*) has shown the benefit of including both non-linear contributions and interactions between SNPS (ascertained via XGBoost) as well as simple additive PRS when explaining heritability in complex phenotypes - do the authors see any potential benefit to such a combined approach with sub-SAGE estimates?*

Answer: Thank you for pointing out this interesting publication to us. We may regard the PRS as a genetic covariate and add this to the XGBoost model for more robust predictions, and then use sub-SAGE to get an interpretation of the effect of the PRS on our outcome. This would be very interesting, since the PRS could now have a non-linear effect on the outcome. However, if the individual SNPs (that make up the PRS in a linear combination) also are entered into the XGBoost model in addition to the PRS, then the requirement of independent covariates for sub-SAGE would be violated. We have added some discussion about this in the “discussion and conclusion” section.

Reviewer 2:

Comment: The results from synthetic data are not very convincing that the method identifies the most important variables with high confidence. The interpretation of score is difficult and no understanding of the range of the score is provided. The testing dataset helps understand the confidence a bit but in real world identifying a testing data every-time is a big task.

Answer: First we address the comment on whether the method identifies the most important features or not. As the quantity we use for feature importance, namely the ERFC, is based on SHAP values, we argue that we indeed can capture what ***the model*** regards as important. As to why this is the case, we would like to refer to the definition of SHAP values in the section "Shapley-based explanation methods". The more a feature influences the prediction of the model compared to when the feature is ignored, the greater is the absolute SHAP value. The ERFC quantity is further explored in [1], but the main purpose is that the importance of each feature can be compared fairly to each other. The mean absolute SHAP value, another global importance measure, would also suffice for this purpose.

However, the main purpose of the simulation is to show the limitation of using SHAP values instead of SAGE and sub-SAGE values. As it appears in Figure 3, feature 12 seems to be regarded as important when using SHAP values when it is not important in the data generating process. By using sub-SAGE *and* using an independent test data, one can see that feature 12 no longer appears to be important.

Regarding the comment on identifying a test data set, one can always do so by splitting the data you have at hand into data used for *constructing* the model, and data used for *testing* the model. This is what was done in [1]. The disadvantage of the split is that you do have less data available for training, but that is a trade-off with the ability to derive useful knowledge from your model. As well, one would like the data used for making the model, and the model used for testing the model to originate from the same probability distribution.

Comment: The simulations are only performed on a sample size of 16,000 which is a moderate samples size but would not be considered large dataset. Authors should show power, sensitivity and specificity of the method on a large set of simulations to represent real world datasets. Variables for simulations should include combinations of:
Sample sizes
Minor allele frequencies
Noise to Signal Ratio
Penetrance and prevalence of the disease

Answer: We do understand that these types of simulations would provide fruitful knowledge as to how sub-SAGE values would perform in GWAS-specific tasks. However, our approach is not limited to GWAS set-ups, which is the reason why we did not do such extensive simulations, which would also be time-consuming. Also, as we mentioned earlier, the purpose of the simulation was to compare SHAP and SAGE/sub-SAGE values.

Comment: Real world data application: UKBB dataset consists of >400K samples, however authors only used 64000 samples. It is unclear why they chose only this subset. If the point of the method is to perform GWAS, then the new proposed method should be able to run GWAS on as large of dataset as the conventional methods. It would be imperative to run the analyses on all samples and all variants (possibly an imputed GWAS dataset).

Answer: See general answer to reviewers. Additionally, we understand from the discussion and conclusion section that it may appear as if we propose sub-SAGE as an alternative to a GWAS. This was not our intention, and so this part has been edited. What we wanted to communicate here was the necessity of a multiple testing procedure if several features are tested simultaneously, such as in a GWAS.

Comment: Non-genetic features such as age, sex, ancestry are among the most important features in the real world analyses. However, no interpretation is provided on the significance of known SNPs while adjusting for these confounders. A standard GWAS analyses would include adjusting for any covariates, how does this method account for covariates?

Answer: Non-genetic features are indeed included in our model as explained in the section "Application on genetic data using the UK Biobank resource", and as described in [1], however we must admit not clear enough in this manuscript. In a classical GWAS set-up using a generalized linear model (GLM), confounders are accounted for by simply adding the confounders as variables/covariates in the GLM, such as principal components for adjusting for population stratification. By including non-genetic, possibly confounder, covariates in a non-linear regression model, it is tempting to believe that unwanted confounder effects are also reduced at least to a certain degree such as for GLMs, even though there is no extensive work on this. This is the reason why we do not claim this explicitly in the paper as well.

In [1] we explain that we limit population stratification and cryptic relatedness by restricting to unrelated White European individuals. In addition, extensive cross-validation was used to limit spurious effects for instance due to population stratification and cryptic relatedness.

This information was not written previously, and we understand this should be included. We have therefore added a few sentences about this in the revised version of our paper.

Comment: Standard GWAS methods assume an additive model and are most powered at identifying additive effects. An improvement over existing GWAS methods would be to identify non-additive effects. Is this method able to identify only known signals or any novel signals as well for BMI that might help elucidate the genetic underpinnings of obesity more than standard GWAS fro UKBB? The previous method published in BMC Bioinformatics focussed on GXG and GXE, does this method also identify those effects, unclear from the manuscript?

Answer: One of the major advantages of SHAP and SAGE values is that they can be applied one any input-output model, linear of non-linear, parametric or non-parametric, which is different from using *p*-values in a linear (or logistic) regression model to test a regression parameter for a particular feature. In non-parametric models such as tree ensemble models, this is not the case. As SHAP and SAGE can be used on non-linear models, non-linear effects can be detected as well.

For those that may not be experienced with the concept of Shapley-based frameworks, we understand that this very important point about detection of non-linear effects was not sufficiently explained in the manuscript. We have therefore added information about this in the "Shapley-based explanation methods" section.

Regarding interactions, it is true that we in this paper restricted ourselves to *marginal* effects, that is the total effect of a feature including the contribution of the feature itself, as well as interactions effects the feature may be involved in. As in [1], similarly to SHAP interaction values, sub-SAGE could in principle be extended to sub-SAGE interaction values. How to incorporate that in an efficient way would be interesting future research work.

We see that these thoughts were not properly discussed in the paper, and we have therefore added a paragraph about this in the Discussion and conclusion section.

Reviewer 3:

Major:

Comment: The ability to scale the algorithm to high-dimensional data relies on considering the pair-wise feature interaction and marginal feature contribution in sub-SAGE. While this is a reasonable choice, a systematic analysis of the loss of power due to such an approximation is missing. The comparison of sub-SAGE and SAGE in Table 2 is done under a simulation that assumes only pair-wise feature interactions.

Answer: We acknowledge the fact that the comparison between SAGE and sub-SAGE when the true relationship only consists of pairwise interactions is not sufficient to investigate the difference in value between sub-SAGE and SAGE. We have therefore emphasized this in the section "Proof of concept - With known underlying data generating process", see end of page 13.

Comment: One contribution the paper claims is the development of an efficient algorithm for global feature importance uncertainty estimation under the tree structure. An alternative approach to achieve the same goal is to instead estimate instance-wise feature attribution values using Tree-SHAP, approximate the global feature importance using the expectation of individual feature attribution estimates, and then use the bootstrap similarly to estimate uncertainty. (Covert et al. have justified the equivalency between these two approaches). The authors should clearly state the advantage of computing global feature importance directly compared to calculating it with the aggregated instance-wise feature importance.

Answer: We mention in the Introduction:

*"As such, SHAP attributes feature importance as they are perceived by the **model**. The more recently introduced Shapley additive global importance (SAGE) is also based on the Shapley decomposition, but attributes feature importance by a global decomposition of the model loss across a whole data set [16]. The SAGE framework thus provides an explanation of the influence of the features taking into account **not only the model**, but also **implicitly the data via the loss function**, thus encapsulating that the model might not be – and most likely is not – a perfect description of the data.",*

and also when we introduce SAGE we write:

"*The use of the loss function in the SAGE definition also ensures that the feature importance is not only based on the **model**, as for the SHAP value, but also on the data itself.*"

The purpose of these arguments is to advocate the use of SAGE instead of SHAP. The reason is that SHAP values are based solely on the model itself, no matter how bad it is. Therefore, a global feature importance value based on Tree-SHAP will also only rely on the output of the model. On the other hand, SAGE will not only take into account the model, but also the importance of the feature with respect to the *loss function,* which again depends on *the data*.

We can see this argument may not be as visible as we wanted, so we have emphasized the argument in the discussion and conclusion section.

Comment: A more general question for the authors is to demonstrate the usefulness of the uncertainty estimates. One natural application is to perform feature selection. If the intention of the uncertainty estimation is for feature selection purposes, it would be valuable to compare it with some commonly used feature selection techniques in the area. Additional analyses and discussion of the utility of uncertainty estimates would strengthen the manuscript.

Answer: The main purpose of quantifying uncertainty estimates of the importance of each feature is perhaps most easily seen if the results from the model are to be investigated further within genomics or medicine. For instance, if a potential discovery of a drug relies on results from a black-box machine learning model, we would need to understand how likely the results are and how certain we are about the results, based on prior knowledge as well the possibility to interpret the machine learning model. We have emphasized this even more strongly in the introduction. We have now added a citation to a paper published in *Nature Machine Learning* that addresses the importance of explainable AI (XAI) within drug discovery.

For the purpose of feature selection, even sub-SAGE would not be feasible in most cases if the number of features gets too large due to running time. Then it would be better to use SHAP values as we also do in our *ranking process* introduced in [1]. Sub-SAGE would be more appropriate as taking the role of p-values to infer the true importance of a feature in a non-linear "black box" machine learning model.

One could question whether information of feature importance would already be available when doing a multiple testing procedure where each feature is tested using a typically a linear model only including the feature itself as well as global covariates to adjust for confounding for instance using the score test (as in a GWAS). However, the importance of the feature in this case would not account for non-linear effects as well as possible interaction effects it may be involved in. However, for a model including a large number of features together, one is more likely to capture at least the most important interactions, even though we agree in this case that we restrict ourselves to pair-wise interactions.

Minor:

1. The notation $Y(\boldsymbol{x})$ relates to the true relationship between features $\boldsymbol{x}$ and response of interest y including stochastic noise. The notation $\hat{y}(\boldsymbol{x})$ relates to an estimate of $E[Y(\boldsymbol{x})]$, namely the expected response y given features $\boldsymbol{x}$.

2. Thank you for noticing and corrected.

[1] P. V. Johnsen, S. Riemer-Sørensen, A. T. DeWan, M. E. Cahill, and M. Langaas, 'A new method for exploring gene–gene and gene–environment interactions in GWAS with tree ensemble methods and SHAP values', *BMC Bioinformatics*, vol. 22, no. 1, p. 230, May 2021, doi: 10.1186/s12859-021-04041-7.

[2] I. Covert, S. Lundberg, and S.-I. Lee, 'Understanding Global Feature Contributions With Additive Importance Measures'. arXiv, Oct. 27, 2020. Accessed: Nov. 20, 2022. [Online]. Available: http://arxiv.org/abs/2004.00668

[3] D. E. Reich *et al.*, 'Linkage disequilibrium in the human genome', *Nature*, vol. 411, no. 6834, Art. no. 6834, May 2001, doi: 10.1038/35075590.

[4] M. J. Sillanpää, ‘Overview of techniques to account for confounding due to population stratification and cryptic relatedness in genomic data association analyses’, *Heredity*, vol. 106, no. 4, Art. no. 4, Apr. 2011, doi: 10.1038/hdy.2010.91.

[5] J. Grau, I. Grosse, and J. Keilwagen, ‘PRROC: computing and visualizing precision-recall and receiver operating characteristic curves in R’, *Bioinformatics*, vol. 31, no. 15, pp. 2595–2597, Aug. 2015, doi: 10.1093/bioinformatics/btv153.
