# Supplementary material for: Inferring feature importance with uncertainties with application to large genotype data
Source: PLoS Comput Biol. 2023 Mar 14;19(3):e1010963. doi: 10.1371/journal.pcbi.1010963 (PMC10038287; doi:10.1371/journal.pcbi.1010963)
Supplement: Attachment — Submitted filename: Response to reviewers - Minor revision.pdf [file pcbi.1010963.s003.pdf]

Response to reviewer comments regarding minor revision of PCOMPBIOL-D-22-01012R1

Again, we are grateful for the constructive comments by the reviewers. Below, is a detailed answer to the comments from Reviewer #3. We refer to edits, and therefore line numbers in the manuscript version including highlights of changes made in the manuscript, from here on denoted the *highlighted version.*

Comment 1*: In lines 472-475, the authors discussed that the threshold and multiple testing procedure is needed to control the false positive rate. The authors should further expand this part since how to set the threshold and demonstrate calibration is not directly clear from the estimated uncertainty computed using the sub-SAGE approach, but a crucial step in GWAS.*

Answer: Thank you for this comment, we agree this part is not sufficiently clear where we may leave the impression as to how to go forward. However, in this paper we chose to not focus on how to construct hypothesis tests for inference, but rather how to be able to construct an estimated percentile interval via bootstrapping. It is however possible to construct a hypothesis testing procedure, provided the ability to use the percentile method for estimating the uncertainty in the Sub-SAGE value.

We have now expanded this part by defining the null hypothesis, namely that Sub-SAGE value is less than or equal to zero, as well as *one* procedure for rejecting the null hypothesis. This is done by constructing a one-sided $(1-\alpha)100\%$ bootstrap percentile interval, such that the lower bound of the interval is the estimated α percentile and reject the null hypothesis if the lower bound is *greater* than zero. However, such a construction like this, based on an estimated confidence interval often sacrifices statistical power as discussed in . We will leave it as future research as to alternative hypothesis testing procedures that may be more powerful than the method we provided.

Regarding the multiple testing procedure, this is another case where we chose to not focus on how to exactly deal with this, but rather emphasize that this would need to be taken care of if the inference will be based on several features simultaneously.

However, we have some thoughts how we envision this to be done, and what to have in mind: In this case it would not be based on hundreds of thousands of features such as in a GWAS, as the model will only be based on a small subset of features, say including hundreds or a few thousands of features. A practical example would for instance be to test the top 10 or top 100 features that are most promising according to some other, more untrustworthy, quantity such as the ERFC we are using in this paper. Therefore, the necessary significance level for each test would not be as small as in a GWAS, and for instance equal to 5×10^{-4} to ensure the familywise error rate (FWER) does not exceed 0.05 when doing 100 tests and applying the Bonferroni correction known to be a conservative method. If the top N features are tested from a ranked list, and many of these features truly are important, Bonferroni correction is particularly known to be conservative (FWER is controlled at $\pi_o\alpha$ where π_o is proportion of true null hypotheses). In this case, other approaches such as Holm's method [1] are more appropriate. Another approach is to use the *false discovery rate* instead which is less strict with respect to control of the false positive rate.

However, as we have not investigated this enough, we choose to leave this as an open question as to how multiple testing should be accomplished in this case.

Comment 2*: In table 3, it would be good to run linear models on the selected feature, compute their feature coefficient and the corresponding p-values and discuss how much benefit we gain from computing feature importance in a nonlinear way using sub-SAGE.*

Answer: Regarding Table 3, this shows the computed *ERFC* quantities based on SHAP values, as we explain in the paper are not as trustworthy as SAGE or Sub-SAGE values. So, with respect to comparison, Table 3 could be used to compare ERFC with *p*-values from a linear model, or a generalized linear mixed model to see the benefit of ERFC compared to *p*-values. However, this was the major goal to show in [2], and we rather want to refer the reader to this paper regarding this question. For instance, the approach in [2] that creates the ranked list of features used in this paper are compared with the generalized linear mixed model BOLT-LMM [3] based on *p*-values. See Figure 9, indicating stronger predictive models based on the approach introduced in [2] than using a ranked list based on *p*-values from BOLT-LMM.

To make it clearer that Sub-SAGE estimates should not be seen as an alternative to the normal procedure in a GWAS, we have made some edits (see lines 162-171 in the highlighted version) where we emphasize that given hundreds of thousands of features, we need to first filter which features to investigate further, for instance via a ranking procedure. Here, we add that this ranking may well originate from a regular GWAS procedure with ranking based on the computed *p*-values. However, an alternative procedure is the one mentioned in [2].

The purpose of Sub-SAGE here is to investigate more thoroughly whether the feature importance quantified by ERFC is trustworthy or not. As we have mentioned before, ERFC will be based on what the constructed model itself regards as important, and therefore heavily dependent on the model uncertainty. Using Sub-SAGE, features that seemingly appear important based on the ERFC quantity, can be revealed to in fact not be so. The simulation example in this paper is an example of this scenario concerning feature "x12", see Figure 2., and compare with Figure 3.

We could do as suggested, namely run a logistic model, and compute *p*-values and compare, akin to what we did in [2], however in this case for interaction effects. However, in the paragraph discussing the SNPs in Table 3, the purpose was to inform about previous knowledge about these SNPs related to obesity. Therefore, from the SNPs given in Table 3 not mentioned before, namely rs489693, rs1488830, rs10913469 and rs2820312, we have chosen to write the calculated *p*-values of the SNPs based on the PheWeb platform [4] which is openly available via the link https://pheweb.org/UKB-TOPMed/. Here a generalized linear mixed model, SAIGE, is used based on TopMed imputation [5].

Comment 3*: In line 398, the SNP rs2820312 exclusively detected by sub-SAGE need further analysis. For example, have any other SNPs in the LD block of SNP rs2820312 been previously detected to be associated with obesity?*

Answer: The SNP rs2820312 referred to in line 398 was with respect to Table 3, where the ERFC is computed as feature importance quantity, and not Sub-SAGE. Even though the ERFC-value for this particular SNP is quite large, we can later see in Figure 4 that the corresponding Sub-SAGE value is estimated to be very small, if even larger than zero. The 95 % bootstrap percentile interval further included the value zero. That is why we in the manuscript are careful with denoting this as a finding.

Using a generalized linear mixed model in UK Biobank from the PheWeb platform explained above, the computed *p*-value for this SNP is 1.1×10^{-2}. The SNP rs2820312 is connected to the LMOD1 gene (https://www.ncbi.nlm.nih.gov/snp/rs2820312), a region in the genome where nearby SNPs, as far as we know, have not been previously statistically associated with obesity. However, as we previously wrote, the SNP is statistically associated with hypertension from the PheWeb platform, and further the analysis in [6] indicates that the LMOD1 gene is linked with pulmonary arterial hypertension . It is also shown that obesity is a strong risk factor to hypertension [7]. So, the apparent small, if any, association between the SNP rs2820312 and obesity might only be because of the association between obesity and hypertension.

As the focus of this paper was the computation of Sub-SAGE, its advantages, and the possibility of inference in association analysis, and not causal analysis, we choose to not spend too much time on this in the manuscript, but rather want to guide the reader to make their own reflections about the results. See edits in line 410-412.

Comment 4: *In lines 450-454, the authors described several sources of the uncertainty of the Sub-SAGE uncertainty estimate. However, another important source of uncertainty, the model uncertainty, needs to be mentioned. I suggest the authors add it to the text.*

Answer: We thank the reviewer for pointing out that model uncertainty should be discussed more. Implicitly we discussed about model uncertainty when we explained the disadvantages of SHAP values, namely that the trustworthiness of the explanations from the SHAP values depend entirely on the model uncertainty. It is here we argue that SAGE and Sub-SAGE values can reveal false positive effects when SHAP values cannot, since Sub-SAGE does not depend entirely on the model, effectively reducing the chance of false positives (we have made this clearer in the discussion, see line 446). However, what we did not discuss is how model uncertainty can affect the interpretation of Sub-SAGE negatively. Indeed, with respect to the statistical power for inference of the Sub-SAGE value, the probability of correctly rejecting the null hypothesis of no feature importance, will depend on the model uncertainty. Assume a feature indeed affects the response. Then, the more the model has captured the true relationship between the particular feature and the response, the greater the probability that the null hypothesis will be correctly rejected. See lines 494-496 in the highlighted version for edits regarding this.

Final remarks:

Regarding choice of subsets to choose in Sub-SAGE, and alternatives. We have added another option for how to set the restricted number of subsets, see line 455-457, namely by sampling from the same probability distribution as is done in the Kernel SHAP method [8], where subsets with the largest Shapley weights are most likely to be sampled.

Finally, we agree with Reviewer #3 that the advantage of sub-SAGE on actual genetic applications still has room for improvement. We hope however that the methods applied in this paper can be of inspiration with respect to how to explain trustworthy, including uncertainty estimation, genetic effects based on a non-linear machine learning model.

[1] J. J. Goeman and A. Solari, ‘Multiple hypothesis testing in genomics’, *Statistics in Medicine*, vol. 33, no. 11, pp. 1946–1978, 2014, doi: 10.1002/sim.6082.
[2] P. V. Johnsen, S. Riemer-Sørensen, A. T. DeWan, M. E. Cahill, and M. Langaas, ‘A new method for exploring gene–gene and gene–environment interactions in GWAS with tree ensemble methods and SHAP values’, *BMC Bioinformatics*, vol. 22, no. 1, p. 230, May 2021, doi: 10.1186/s12859-021-04041-7.
[3] P.-R. Loh *et al.*, ‘Efficient Bayesian mixed model analysis increases association power in large cohorts’, *Nat Genet*, vol. 47, no. 3, pp. 284–290, Mar. 2015, doi: 10.1038/ng.3190.
[4] S. A. Gagliano Taliun *et al.*, ‘Exploring and visualizing large-scale genetic associations by using PheWeb’, *Nat Genet*, vol. 52, no. 6, pp. 550–552, Jun. 2020, doi: 10.1038/s41588-020-0622-5.
[5] D. Taliun *et al.*, ‘Sequencing of 53,831 diverse genomes from the NHLBI TOPMed Program’, *Nature*, vol. 590, no. 7845, Art. no. 7845, Feb. 2021, doi: 10.1038/s41586-021-03205-y.
[6] S. Sahoo *et al.*, ‘MEF2C-MYOCD and Leiomodin1 Suppression by miRNA-214 Promotes Smooth Muscle Cell Phenotype Switching in Pulmonary Arterial Hypertension’, *PLoS One*, vol. 11, no. 5, p. e0153780, May 2016, doi: 10.1371/journal.pone.0153780.
[7] J. E. Hall, J. M. do Carmo, A. A. da Silva, Z. Wang, and M. E. Hall, ‘Obesity-Induced Hypertension’, *Circulation Research*, vol. 116, no. 6, pp. 991–1006, Mar. 2015, doi: 10.1161/CIRCRESAHA.116.305697.
[8] K. Aas, M. Jullum, and A. Løland, ‘Explaining individual predictions when features are dependent: More accurate approximations to Shapley values’, *Artificial Intelligence*, vol. 298, p. 103502, Sep. 2021, doi: 10.1016/j.artint.2021.103502.
